# TORCH: Toronto Outcome Research in Child Health - enhancing evidence based outcomes selection in pediatric research

**DOI:** 10.1186/1745-6215-16-S3-P2

**Published:** 2015-11-24

**Authors:** Mufiza Z Kapadia, Martin Offringa

**Affiliations:** 1 Toronto Outcomes Research in Child Health (TORCH), SickKids Research Institute, Toronto, Canada

## Background

Children’s responses to medical treatments differ significantly from adults. Appropriately selecting and measuring child and family relevant outcomes when designing pediatric clinical trials is important for decision making with regards to the health of the child. However, outcomes used to measure an intervention’s effectiveness in current pediatric clinical trials often lack child and family relevance, are heterogeneous across and within child health ages and diseases, and are not adequately measured with validated instruments. Furthermore, involvement of patients (children) and their proxies (usually parents) in outcomes selection is minimal. Inconsistent use of outcomes and outcomes measurement in pediatric clinical trials impairs the synthesis of evidence in systematic reviews and leads to outcome reporting bias. This high variability in outcome selection and measurement has led to a situation where child health decisions on treatment of children lack the appropriate underpinning evidence, and a subsequent inability to reach a consensus on the effectiveness and safety of a treatment. Recently, outcome selection initiatives in the general population such as OMERACT and COMET advocate homogeneity of methodology for outcome selection and measurement in trials.

## Method

Toronto Outcome Research in Child Health (TORCH) is an exciting new collaborative initiative that develops and employs existing and new evidence-based methods for improving outcomes selection and measurement in cohort studies and trials in children.

## Results and discussion

The TORCH platform raises awareness on the importance of meaningful outcomes selection and measurement in children; provides methodology to select measure and report truthful, discriminative and feasible outcomes in child health research; and supports engagement with research ethics boards, funders, journal editors and regulators to critically appraise outcomes selection, measurement and reporting in any child health research. The new TORCH platform will facilitate the translation of knowledge from the literature to bedside care, thereby improving child health outcomes while reducing the burden on the healthcare system.

